# Protein-Energy Wasting and Mortality in Chronic Kidney Disease

**DOI:** 10.3390/ijerph8051631

**Published:** 2011-05-19

**Authors:** Alice Bonanni, Irene Mannucci, Daniela Verzola, Antonella Sofia, Stefano Saffioti, Ezio Gianetta, Giacomo Garibotto

**Affiliations:** 1 Division of Nephrology, Dialysis and Transplantation, Department of Internal Medicine, Azienda Ospedale Università San Martino, Genoa University, Viale Benedetto XV 6, Genoa, Italy; E-Mails: alice.bonanni@libero.it (A.B.); ire28182@yahoo.it (I.M.); daverz@libero.it (D.V.); antonella.sofia@unige.it (A.S.); stesaf@unige.it (S.S.); 2 Department of Surgery, Azienda Ospedale Università San Martino, Genoa University, Largo R. Benzi, Genoa, Italy; E-Mail: gianetta@unige.it

**Keywords:** protein-energy wasting, malnutrition, chronic kidney disease, cardiovascular risk, skeletal muscle

## Abstract

Protein-energy wasting (PEW) is common in patients with chronic kidney disease (CKD) and is associated with an increased death risk from cardiovascular diseases. However, while even minor renal dysfunction is an independent predictor of adverse cardiovascular prognosis, PEW becomes clinically manifest at an advanced stage, early before or during the dialytic stage. Mechanisms causing loss of muscle protein and fat are complex and not always associated with anorexia, but are linked to several abnormalities that stimulate protein degradation and/or decrease protein synthesis. In addition, data from experimental CKD indicate that uremia specifically blunts the regenerative potential in skeletal muscle, by acting on muscle stem cells. In this discussion recent findings regarding the mechanisms responsible for malnutrition and the increase in cardiovascular risk in CKD patients are discussed. During the course of CKD, the loss of kidney excretory and metabolic functions proceed together with the activation of pathways of endothelial damage, inflammation, acidosis, alterations in insulin signaling and anorexia which are likely to orchestrate net protein catabolism and the PEW syndrome.

## 1. Protein Energy Wasting (PEW) in CKD: Clinical Implications

In spite of the advancements in current techniques of renal replacement therapy, mortality levels remain high in patients with CKD [1]. This increase in mortality is not only limited to dialysis patients, but includes the whole ranges of GFR during the progression of CKD [2,3] and is mainly due to cardiovascular disease (CVD) and, at advanced stages, to infections [4]. The prevalence of protein energy wasting (PEW), a condition of loss of muscle and visceral protein stores not entirely accounted for by inadequate nutrient intake [5] increases progressively along with the loss of residual renal function and is high in dialysis patients [5].

Uremia-induced alterations in protein metabolism and gastrointestinal tract function can result in poor nutritional status, which in turn increases the risks of CVD and infection. The classical risk factors for CVD (such as old age, lifestyle, smoking, hypertension, dyslipidemia, diabetes, left ventricular hypertrophy, hearth failure) are over-expressed in CKD patients, partly because of the clinical characteristics of the CKD population (which consists mainly of old subjects, with many of them affected by CVD and type II diabetes) [6]. However, the excess of cardiovascular risk related to CKD may be due in part to a higher prevalence of non-traditional risk factors peculiar to CKD which *per se* may promote endothelial dysfunction and/or atherogenesis [1].

An example of the relevance of non-traditional risk factors is offered by the phenomenon of the “reverse epidemiology” in dialysis patients [7]. While in the general population a high body mass index (BMI; Kg/m^2^) is associated with an increased cardiovascular risk and with all-cause mortality, in dialysis patients the effect of overweight or obesity is paradoxically in the opposite direction, with a higher BMI leading to an improved survival [7]. The “reverse epidemiology” phenomenon involves also several other traditional risk factors, such as blood pressure and serum concentrations of cholesterol, homocysteine, and creatinine [8]. In addition, during the course of progressive decline in renal function, the profile of risk for death may change because the occurrence of others risk factors, such as progressive wasting [9], and inflammation [10], which may play an increasing role and may outweigh the effects of traditional risk factors. Chmielewski *et al.* [11] recently showed that apoB/apoA-I ratio is associated with greater survival in hemodialysis (HD) patients only in the short term (1-year mortality).Similar data were obtained in another study [12], where the reverse association between hypercholesterolaemia and all-cause mortality declined gradually after the first year of follow up. This is probably due to the temporal impact of competing risks.

Liu *et al.* [13] recently postulated that the paradoxical relationship between cholesterol level and mortality could be explained by the effect of the presence of the complex malnutrition-inflammation (defined as BMI < 23 kg/m^2^ or C-reactive protein > 10 mg/L) in the dialysis population. Recently, Contreras *et al.* [14] assessed the prevalence of malnutrition-inflammation and its modifying effects on the risk-relationship of cholesterol levels with subsequent CVD events in African American with hypertensive CKD. They showed that the hazard ratio for the primary CVD outcome increased as total cholesterol increased in subjects without malnutrition-inflammation, whereas it tended to decrease in subjects with malnutrition-inflammation. It should be considered that the phenomenon of “reverse epidemiology” is not exclusive of renal patients, but is also observed in sarcopenia of aging [15] and in chronically wasted patient groups affected by chronic heart failure, HIV infection or cancer [16] suggesting that the effects of wasting may overcome those of traditional risk factors for cardiovascular disease.

The aim of this work is to review the mechanisms responsible for PEW, while providing a summary of the non-traditional factors which increase the cardiovascular risk in CKD patients. The mechanisms underlying the causes of the wasting syndrome are beginning to be understood and include the loss of kidney metabolism and function as well as the activation of pathways of endothelial damage, inflammation, acidosis, altered intracellular IGF-1 and insulin signaling. These factors overlap with those already operating in ageing and in comorbid conditions, such diabetes and sepsis, and are likely to orchestrate the PEW syndrome.

## 2. Biomarkers/Proxies for Malnutrition and Mortality in CKD

Several biomarkers have been associated with worse outcomes in CKD and dialysis patients. Among these, those of PEW appear to be the strongest predictor of survival [9]. Lower serum albumin [17], prealbumin [18], cholesterol [13], serum transferrin [18], creatinine [19] and bicarbonate [20] levels are associated with mortality in dialysis patients. Other biochemical markers that are directly or indirectly linked to PEW and outcomes include various hormones such as testosterone [21], leptin [22], visfatin [23], adiponectin [22] and thyroid hormones [24,25]. The mechanisms of action responsible for the adverse outcomes associated with PEW markers remain unclear: it is likely that a combination of factors are responsible, rather than a single etiologic mechanism [9].

### 2.1. Hypoalbuminemia

Hypoalbuminemia is the most commonly used surrogate of PEW in dialysis patients and has a strong association with increased mortality [17] and morbidity [26]. Hypoalbuminemia is associated with development of *de novo* and recurrent cardiac failure in HD and CAPD patients [27]. The use of serum prealbumin has been advocated as a better surrogate of nutritional status than albumin in dialysis patients [28]. A confounding factor is that serum albumin and prealbumin are also negative acute phase reactants and their serum levels are profoundly affected by the presence of an inflammatory response. It is already known from studies in 1940s (the “Minnesota Experiment”), that albumin levels are extraordinarily preserved, while fat an muscle mass is lost, when nutrient intake is curtailed [29]. Also CKD patients compliant with a restricted protein and energy intake do not show a remarkable decrease of serum albumin levels [30,31]. In dialysis patients, hypoalbuminemia could also be favoured by the loss of amino acids and/or protein during renal replacement therapy [32]. Therefore, it is not clear whether the negative clinical outcome in advanced CKD patients associated with hypoalbuminemia is a reflection of nutrition or of the inflammatory response or both. It is also not clear whether the relationship between hypoalbuminemia and increased mortality in dialysis patients stems from the intrinsic effects of albumin, or hyopoalbuminemia represents the occurrence of ongoing events associated with increased mortality risk. Low albumin may be associated with hypercoagulable states [33], and increased blood viscosity [34]. Moreover, low oncotic pressure may adversely affect water shift between the intravascular and interstitial space. Albumin also has important roles as a scavenger of free radicals, is a binding agent for toxic compounds and a carrier for a wide variety of drugs and hormones [35]. Reduced albumin binding of drugs and endogenous ligands is a feature of uremia [36].

### 2.2. Oxidative Stress

Oxidative stress results from an imbalance between oxidant generation and anti-oxidant defense mechanisms, leading to cell and tissue injury. It is consistently accepted that in CKD patients a deficiency in the antioxidant systems combines with an increase in pro-oxidant activity owing to advanced age, diabetes, chronic inflammation, sepsis and the bio-incompatibility of dialysis membranes and solutions. Nguyen-Khoa *et al.* [37] found a direct correlation between lipid and protein oxidation markers and CRP levels; in addition, plasma alpha-tocopherol resulted inversely correlated with CRP levels and with the duration of dialytic treatment [37]. The accumulation of AGEs observed in CKD could be partly due to a reduced renal clearance of these compounds, but also to an increased *de novo* generation, stimulated by oxidative stress and inflammation. AGEs could play an important role in the development of atherosclerosis, by stimulating the activation of mononuclear cells (thus inducing endothelial dysfunction) and also by modifying LDLs, which become less able to be cleared by the LDL receptors [38]. Moreover, the interaction between AGEs and their receptors (RAGEs), could lead to the generation of intracellular oxidative stress and inflammatory mediators through the NF-kB pathway [39]. These mechanisms could explain the role of AGEs in the high frequency of CVD and increased all-cause mortality. Nakashima *et al.* [40] found that both sRAGE and the RAGE ligand S100A12 are elevated in CKD patients. However, while a direct correlation was observed between S100A12 and CRP, IL-6 and cardiovascular mortality, sRAGE resulted negatively associated with CRP and didn’t predict mortality.

Advanced oxidation protein products (AOPPs) are a class of dityrosine-containing protein products, which arise from the reaction between chlorinated oxidants and plasma proteins. AOPPs could play a role in the progression of CKD, since they induce podocyte apoptosis through the activation of NADPH-oxidase [41]. In addition, AOPPs can trigger the phagocyte nicotinamide adenine dinucleotide phosphate (NADPH) oxidase and myeloperoxidase (MPO)-dependent activities [42]. Descamps-Latscha *et al.* [43] found a direct correlation between CRP, fibrinogen, AOPP levels and incident first occlusive atherosclerotic cardiovascular events in CKD patients. Some possible errors in the determination of AOPPs in plasma derive from their UV detection since triglycerides and other particles determining turbidity may interfere in their quantitation and cause their overestimation. Valli *et al.* [44] showed a strong correlation between triglycerides and AOPPs not only in CKD patients, but also in healthy controls.

### 2.3. Microinflammation

Several visceral proteins and hormones which decrease in blood in response to malnutrition (such as albumin, prealbumin, transferrin, retinol binding protein and IGF-I) are also negative acute-phase proteins and their liver synthesis is depressed by inflammation. Several investigators have shown that a significant percentage of chronic hemo- and peritoneal dialysis patients have increased levels of pro-inflammatory cytokines (IL-1, IL-6, TNF alpha) suggesting that a systemic inflammatory response is common in dialysis-treated patients [45,46]. Moreover, cross-sectional studies suggest that inflammation is responsible for serologic and anthropometric evidence of malnutrition [46]. Elevated CRP is strongly associated with cardiovascular mortality [47], so that the association between inflammation and atherosclerosis seems to be particularly strong in dialysis patients. Although precise mechanisms that contribute to the high prevalence of inflammation in CKD are unknown, ROS have been proposed as a potential contributor. On one hand, oxidative stress is able to activate transcriptor factors, such as NF-kB, which regulate inflammatory mediator gene expression. On the other hand, chronic inflammation may cause increased oxidative stress, thus creating a vicious circle in the determination of cardiovascular risk in CKD patients.

Notably, CRP and IL-6 levels increase progressively along with the decline of glomerular filtration rate in CKD patients [45,48]. These effects could be due to reduced kidney excretory function, since at least some pro-inflammatory cytokines are excreted through the kidney. In addition, haemodialysis may activate an inflammatory cascade, because of the exposure of blood to bioincompatible dialysis membranes and/or backfiltration of lipopolysaccharide through the dialysis membranes [49].

Pro-inflammatory cytokines may cause anorexia, induce resistance to GH and IGF-I [50] and increase energy expenditure [51]. CRP *per se* may induce vascular damage, since CRP formed locally in the kidney reduces nitric oxide production and induces monocyte recruitment and foam cell formation [52].

New inflammatory markers are coming into play. Pentraxin-3 (PTX3), an inflammatory mediator structurally linked to CRP produced by vascular endothelial cells and macrophages in response to pro-inflammatory signals, has been shown to be elevated in haemodialysis patients [53], and has been identified as a novel mortality risk factor in incident dialysis patients and CKD patients, independent of traditional risk factors, CRP and flow-mediated dilation [54,55]. Of interest, fat also seems to be a significative source of PTX3 [56].

Another recently identified biomarker of inflammation in CKD patients is CD14. This molecule is a pattern-recognition receptor that plays a central immunomodulatory role in pro-inflammatory signaling in response to a variety of ligands, including endotoxin. CD14 protein is present both in soluble (sCD14) and membrane-bound forms. Raj *et al.* [57] showed an association between sCD14 levels and the presence of PEW in hemodialysis patients, probably due to cytokine activation.

## 3. Effect of Body Composition on the Risk of Mortality of CKD Patients

The protective effect of large body size in dialysis patients may be associated with higher adipose tissue and fat reserves. This appears to be somewhat contradictory, since adipose tissue, especially visceral fat, has pro-inflammatory properties. As an alternative explanation, the “protective” effect of BMI might be related to fat-free mass. Even though fat tissue is related to inflammation in the general population, in dialysis patients it may be associated with relatively greater secretion of anti-inflammatory cytokines, such as adiponectin, than pro-inflammatory molecules [58]. There is no difference in plasma inflammatory markers (IL-6, TNF-α, and CRP) in individuals with different proportions of body fat [59]. A limited number of studies have examined the relative contributions of fatty mass (FM) and lean body mass (LBM) to clinical outcomes in CKD patients who undergo maintenance haemodialysis [60,61]. Some of these studies have suggested that the protective effect of high BMI against mortality is related to higher FM [62].

Recent findings suggest that the type and the distribution of fat rather than its total amount play a role in the determination of risk [63]. Particularly, abdominal fat accumulation is a mortality risk factor, since it is a source of pro-inflammatory adipokines [64]. Both leptin and visfatin seem to play a role in CVD and endothelial dysfunction [65,66] and the receptors of these adipokines are highly expressed in atherosclerotic plaques [67,68]. *Ramkumar et al.* [69] however, suggested that the protective effect of BMI in the maintenance haemodialysis population is mostly conferred to those patients with a higher LBM (muscle mass), estimated by urine creatinine content. Huang *et al.* [70] recently reported that low mid-arm muscle circumference (MAMC), a surrogate of LBM, and low triceps-skinfold thickness, an indicator of FM, were each associated with higher all-cause mortality in haemodialysis patients. Higher FM in both sexes and higher LBM in women appear to confer a protective effect. However, the survival advantage of FM appears to be superior to that of LBM [71].

## 4. Mechanisms of PEW

### 4.1. Altered Amino Acid and Protein Handling by the Diseased Kidney

The human kidney plays a major role in the homeostasis of body amino acid pools. The kidney is the major organ for the disposal of glutamine and proline from the arterial blood, and for the net release of some amino acids such as serine, tyrosine and arginine, which are newly generated within the kidney for export to other tissues [72,73]. The magnitude of the net release or uptake of amino acids by the normal kidney can be understood if one considers that in a 70 Kg man the daily net production of serine is ∼3–4 g, that of tyrosine ∼1 g, that of arginine ∼2 grams. In addition the human kidney plays also a major role in the removal of glutathione [74], and of S-adenosylhomocysteine (SAH) [75]. Besides that, the kidney also releases smaller amounts (<1 g/day) of threonine, lysine and leucine into the systemic circulation [72].

The human kidney plays also a major role in whole body protein metabolism and amino acid oxidation [76,77] as well as in the regulation of plasma levels of several hormones and peptide compounds (*i.e.*, prostaglandins, thyroid hormone, parathyroid hormone, insulin, growth hormone (GH), 1,25 dihydrocholecalciferon), whose concentrations and effects result therefore altered in renal failure.

### 4.2. The Kidney and Methionine Transmethylation

The biological effects of methionine transmethylation, by which the methyl group of S-Adenosylmethionine (SAM) is donated to a large variety of acceptor substrates, is needed for the synthesis of a wide range of compounds such as membrane phospholipids, neurotransmitters, proteins, creatine and hormones [76]. Methylation processes play also a major role in the epigenetic regulation of protein expression and changes in human DNA methylation patterns are an important feature of many diseases, including atherosclerosis and cancer [78]. S-adenosylhomocysteine (SAH) is the by-product of methionine transmethylation and the precursor of homocysteine. Since SAH is a potent feedback inhibitor of most methyltransferases, including the methionine remethylation pathway, this compound plays an essential role in the control of the overall transmethylation rates [76]. Thus, the efficiency of methyltransferase reactions is dependent on efficient tissue removal of SAH [79]. Recent experimental [80] and clinical evidence [81] also suggests that the accumulation of SAH in body fluids, rather than increased homocysteine levels, is associated with vascular disease and tissue damage. Posttranslational modification of proteins, associated with high SAH intracellular accumulation, has been described in patients with CKD [82]. To explore the sites and mechanisms underlying the regulation of circulating SAH levels, we measured plasma SAH across the kidney, splanchnic bed and lung in humans [75]. Our results showed that the human kidney plays a unique role in the removal of SAH from the circulation, indicating that the kidney may have an important role in the control of body transmethylation reactions.

### 4.3. The Kidney and Nitric Oxide (NO) Synthesis

In the endothelium, a central regulatory reaction is the generation of NO from l-arginine, which is controlled by NO synthase. It is of note that the NO production is decreased in CKD patients [83]. There are many causes to explain the NO deficiency. One obvious cause is that limited arginine provision by diseased kidney hinders tissue availability of this amino acid. However, a decrease in arginine levels is rarely observed in CKD patients suggesting the existence of sources of arginine other than the kidney [76]. In patients with advanced renal disease, anorexia could blunt nutritional intake, thus further curtailing tissue arginine pools. NO deficiency could be induced by increased reactive oxygen species (ROS) generation and by decreased tetrahydrobiopterin (BH4), an important cofactor for NOS [84]. In addition, the accumulation in blood fluids of NO Synthase inhibitors, such as asymmetric dimethilarginine (ADMA) could explain the strong association between ADMA and carotid atherosclerosis as wall as with overall and cardiovascular mortality [85]. Initially, it was assumed that the increased plasma ADMA in ESRD reflected loss of renal clearance [86]. However, very little ADMA is excreted unchanged in the urine and the majority is broken down by the enzymes dimethylarginine dimethylaminohydrolase 1 and 2 (DDAH1 and 2). DDAH is widely distributed and is most abundant in the kidney and also highly expressed in the liver [85]. Decreased metabolism by DDAH could be a further cause for ADMA accumulation in CKD patients. ADMA appears to be involved in CKD progression, since elevated concentrations of ADMA are associated with the development of renal fibrosis in animals [87]. These data suggest that in pathophysiological conditions of endothelial dysfunction, the exaggerated endogenous synthesis of ADMA could contribute to CKD progression by favoring hypertension and extracellular matrix synthesis. Short-term reduction of circulating ADMA by hemodialysis is associated with amelioration of endothelial dysfunction in patients with ESRD [88].

### 4.4. Resistance to GH/IGF-1

Resistance to GH is responsible for the defective growth of uremic children. Besides its stimulatory effects on growth and anabolism, circulating GH and IGF-1 levels may play a role in the metabolic response to fasting, by contributing to protein conservation [72]. Growth hormone (GH) resistance is common in uremia and together with resistance to insulin-like growth factor-1 (IGF-1) contributes to muscle wasting. Development of GH resistance in uremia stems from reduced IGF-1 synthesis [89], sensitivity [90] or bioavailability [91], but also to impaired GH signal transduction [92]. Defective intracellular GH signaling may stem from impaired GH stimulated Jak2-STAT5 phosphorylation, which may be the result of inflammation induced SOCS2 expression [92]. In addition, the hepatic resistance to GH-induced IGF-1 expression in uremia arises due to defects in STAT5b phosphorylation and its impaired binding to DNA, processes further aggravated by inflammation [93].

Besides uremia *per se*, the effectiveness of GH might also be attenuated by other factors often found in CKD patients, such as metabolic acidosis [94] and low nutrient intake [95]. We recently observed [96] that the GH acute anabolic response regarding two different GH receptor-downstream pathways (potassium and amino acid metabolism) is overall preserved in patients with advanced CKD, while it is blunted in patients displaying evidence of microinflammation, suggesting a role for inflammatory changes in the regulation of skeletal muscle protein balance.

### 4.5. Low Testosterone

Multiple anabolic signals, which include both an increase in the regenerative potential and protein metabolism are activated by androgens. Testosterone stimulates myoblasts and satellite cell proliferation to promote repair of muscle damage [97]. In addition, testosterone stimulates muscle protein synthesis [98].

Testosterone concentrations decline with aging and chronic disease [99,100] and are correlated to worse survival [101,102] and mortality due to cardiovascular disease [103]. Among men treated with hemodialysis, testosterone concentrations inversely correlate with all-cause and CVD-related mortality, as well as with markers of inflammation, which suggests that hypogonadism may be an additional treatable risk factor for patients with CKD [104,105].

### 4.6. Insulin Resistance and Altered Insulin Signaling

Insulin is a key regulating factor of protein metabolism for the conservation of lean body mass. Even small increases in blood insulin levels, well within the physiological range, are associated with pronounced suppression of protein breakdown [106]. A post-receptor defect in muscle responsiveness to insulin is the cause of insulin resistance with regard to glucose metabolism occurring in CKD patients [72]. However, it is not understood whether insulin resistance regarding glucose metabolism also extends to the antiproteolytic action of this hormone. If so, it would contribute to the muscle wasting which is often found in uremic patients. Bailey *et al.* recently identified a series of abnormal postreceptor signaling changes in the insulin/IGF-1 pathway in muscle of rats with CKD [107]. These included the occurrence of functional abnormalities in the IRS/PI3-K cascade that decrease the phosphorylation of the downstream effector Akt. The low phosphorylated Akt activity has been shown to stimulate the expression of specific E3 ubiquitin conjugating enzymes, atrogin-1/MAFbx and MuRF1, in muscle. Further, a decrease in muscle PI3K activity could activate Bax leading to stimulation of caspase-3 activity and increase protein degradation [107]. These defects which are specific to uremia can overlap with those due to diabetes in patients with diabetic kidney disease. Pupim *et al*. reported that patients with ESRD secondary to DM have an acceleration of loss of lean body mass as compared with nondiabetic CKD patients [108].

Metabolic acidosis is a common complication of advanced CKD and may represent another factor associated with increased insulin resistance. Acidosis increases protein degradation through an upregulation of the ATP-dependent, ubiquitin-requiring pathway [109]. Correction of metabolic acidosis with bicarbonate supplementation has been demonstrated to improve insulin resistance in animal models of uremia as well as in humans [110].

### 4.7. Anorexia

Anorexia, together with nausea and vomiting, is one of the signs of uremic intoxication. In addition to uremia *per se*, other possible causes of anorexia in CKD patients include gastroparesis (diabetic nephropaty), depression and systemic disease (*i.e.*, SLE, congestive hearth failure). Anorexia contributes with inflammatory state to the determination of PEW. Moreover, anorexia is associated with increased mortality risk in hemodialysis patients [111]. Uremia may cause anorexia through the accumulation of toxic molecules, pro-inflammatory cytokines, and middle-size molecules which can inhibit appetite. Cheung *et al.* [112] found that the injection of agouti-related protein (AgRP) in the lateral ventricle of mice with subtotal nephrectomy induced an increase in food intake and body growth, thus suggesting that uremia could cause a defect in the capacity of AgRP to block the melanocortin-4 receptor, MC4-R. Kalantar Zadeh *et al.* [113] showed an association between anorexia and higher concentration of proinflammatory cytokines, hypo-responsiveness to erythropoietin, hospitalization rates and all-cause mortality in hemodialysis patients.

Carrero *et al.* recently found a correlation between appetite, malnutrition, inflammation and outcome in hemodialysis patients [114]. Moreover, they shown a greater susceptibility to inflammation-induced anorexia in men, as compared to women, in agreement with the recent finding in rats of sex specific orexigenic and anorexigenic mechanisms in response to inflammation [115].

## 5. Ageing and Protein Metabolism

In several Western countries, persons over the age 65 are expected to soon become the majority of those who will need renal replacement therapy. Nutritional problems are common in elderly patients with ESRD and contribute to the debility and morbidity in this group of dialysis patients [116]. Low dietary intake and diminished muscle masses are both common in the old individuals and may cause low values of BUN and serum creatinine, even in the presence of advanced renal failure [117].

A decrease in body protein is a major characteristics of ageing [118,119]. It involves mainly muscle proteins, and is associated with decreased muscle strenght and functional impairment. Whole body protein synthesis and degradation are similar in young and elderly adults, when results are expressed per lean body mass [120,121]. However, selected deficits are specific to certain muscle protein components, such as myosin heavy chain and mitochondrial protein [122]. In addition, in elderly subjects a decrease sensitivity of insulin action regarding protein metabolism have been described [123]. These alterations in protein metabolism due to senescence likely potentiate those caused by uremia. Weight loss in older adults is highly predictive of increased morbidity and mortality [124,125]. Several factors contribute to weight loss in older adults. Available data indicate that excess cytokine elaboration may be a critical factor in the induction of involuntary weight loss in older adults. Aging is associated with increased concentrations of TNF-alfa, IL-6, IL1 receptor antagonist, and soluble TNF receptor. Acute phase proteins such as C-reactive protein and serum amyloid-A are also elevated, which suggests the activation of the entire inflammatory cascade.

## 6. Infections

**Figure 1.**
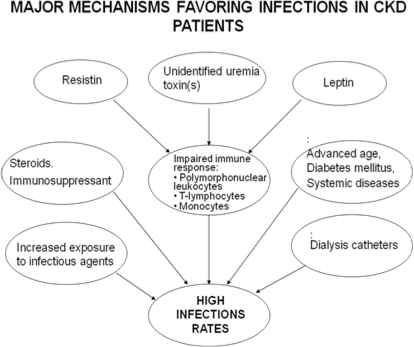
Major mechanisms promoting infections in patients with chronic kidney disease.

Chronic uremia is considered a state of acquired immunodeficiency [126] and CKD patients are at high risk for infection [127]. Among participants in the HEMO Study [128] who died during follow-up, infection was the primary cause of death in 23%. The overall probability of death during an infection-related hospitalization was 15%. Numerous factors, including advanced age, diabetes, hypoalbuminemia, immunosuppressive therapy, dialysis catheters, the dialysis procedure and uremia per se potentially predispose CKD patients to infections (Figure 1). Malnutrition causes defective immune function because of enhanced susceptibility to infections and deficient damage healing [129]. There are nutrients such as arginine and glutamine which can improve the immune response [130–132]. CKD patients are predisposed to zinc [133,134], vitamin B6 (pyridoxine), vitamin C and folic acid deficiencies [135,136], which can lead to alterations in host defense.

The function of polymorphonuclear white blood cells, lymphocytes, and monocytes is altered in ESRD patients [137–139]. Malnutrition, increased intracellular calcium, iron overload, dialysis membranes, and uremic toxins (*i.e.*, circulating factors that inhibit granulocytes) contribute to impaired polymorphonuclear leukocyte function [126,138]. In addition, T lymphocyte [140], monocyte, and monocyte-derived dendritic cell function is also impaired [141]. Leptin, which accumulates in CKD patients, also plays a role in innate and acquired immunity [142,143]. In fact, leptin and its receptor share structural similarities with IL-6, IL-11, and IL-12 [144,145]. Leptin regulates T lymphocyte responses [146] and increases the secretion of several cytokines by endotoxin-stimulated peritoneal macrophages [147]. In addition, leptin induces the expression and secretion of IL-1 receptor antagonist (IL-1Ra) by human monocytes [148] as well as the production of TNF and IL-6 [149]. Recent findings show that leptin positively modulates mononuclear cell survival by interfering with the apoptotic process [150]. More striking, hypoleptinemia characterizing starvation is strictly related to increased susceptibility to infection secondary to malnutrition [146,151]. Thus, leptin is now considered as an adipokine which is capable of linking metabolism and immune homeostasis [151,152]. In addition has been observed that leptin inhibits neutrophil migration in response to classical chemoattractants.

The serum levels of resistin, a 12-kDa protein expressed in inflammatory cells, are increased in patients with CKD and/or diabetes [153,154]. These patients have an increased risk of infections because of an impaired polymorphonuclear leukocyte (PMNL) functions. Cohen *et al.* observed that resistin interferes with chemotaxis and oxidative burst of PMNL and that p38 MAPK, ERK (p44/42) and PI3K are involved in the regulation of PMNL chemotaxis. Resistin inhibits PNMLs at the high concentrations found in serum of uremic patients, but not in concentrations in healthy subjects [155].

## 7. A New Perspective: Accelerated Loss of Myonuclei and Defective Regenerative Potential in the Skeletal Muscle of CKD Patients

Decrease in nutrient intake, metabolic acidosis, physical inactivity, diabetes, and sepsis are conditions associated with CKD which can promote muscle wasting through an increase in protein degradation and or a decrease in protein synthesis [156–160]. In addition, the balance between cell loss and regeneration can hinder muscle size and function. The role of apoptosis is a new concept in the regulation of muscle size and function in physiology and disease [161,162]. During atrophy nuclei are lost by apoptosis, while during hypertrophy new nuclei are added to the fibres from muscle stem cells (satellite cells) [161,162]. A same signal can theoretically cause cell and protein loss, since Akt can mediate the effects of PI3-kinase on different events, such as apoptosis [163–165] and protein catabolism [165]. These effects may be especially injurious in uraemia, where impaired Insulin-like Growth Factor I (IGF-I) signaling causes abnormal protein metabolism in muscle and decreases the proliferation of satellite cells [166]. Zhang *et al.* observed that transcription factors presents in myonuclei, MyoD and myogenin, are reduced in intact and injured muscle of CKD mice. They also found that in satellite cells of injured muscle the expression of myogenic factors mRNAs is impaired in CKD, while it is stimulated in normal injured muscle [166,167].

Recently, we analyzed muscle apoptosis and myostatin mRNA and their related intracellular signals in rectus abdominis samples obtained from CKD patients scheduled for peritoneal dialysis. Apoptotic loss of myonuclei was 3–5 fold increased. In addition myostatin and IL-6 gene expressions were enhanced by ∼50%, while levels of IGF-I mRNA were lower than in controls. Phosphorylated JNK and its downstream effector, p-c-Jun, were upregulated, while p-Akt was markedly downregulated. Multivariate analysis models revealed p-Akt and IL-6 to contribute individually and significantly to the prediction of apoptosis and myostatin gene expression, respectively. These findings demonstrate the occurrence of the activation of multiple pathways to promote muscle atrophy in skeletal muscle of CKD patients. Both apoptosis of myonuclei and myostatin are upregulated. However, they appear to be associated with different signals, suggesting that their intracellular pathways are differently regulated in CKD patients [168].

Changes in muscle metabolism of specific amino acids induced by uremia could impair muscle regeneration. In patients with CKD, a reduced release of valine and leucine from muscle is likely responsible for their reduced levels in blood [169]. It has been supposed that an increased muscle degradation of valine, probably due to metabolic acidosis [165] and /or an impaired glucose utilization, accounts for the low release of this amino acid from peripheral tissues. Studies in rats and humans with CKD indicate that the correction of metabolic acidosis raises both plasma and muscle BCAA levels by decreasing the transamination and decarboxylation in muscle [170,171]. During the course of CKD, abnormalities due to decreased nutrient intake overlap those caused by acidosis, and the declining plasma valine levels have been reported as an index of poor nutrition and reduced lean body mass [172]. It is of note that leucine cooperates with IGF-1 in stimulating the activation of myogenic satellite cells. These cells are responsible for muscle regeneration in different situations, such as damage-induced muscle loss, aging and progressive neuromuscular diseases. The leucine-induced activation of satellite cells is obtained through the mammalian target of rapamycin (mTOR) signaling, one of the main pathways responsible for protein synthesis and cells proliferation. These effects appear to be due to Beta-hydroxy-beta-methilbutyrate (HMB), a leucine catabolite, which may induce myoblasts proliferation, Akt phosphorylation, and prevent muscle wasting *in vitro* [173].

**Figure 2.**
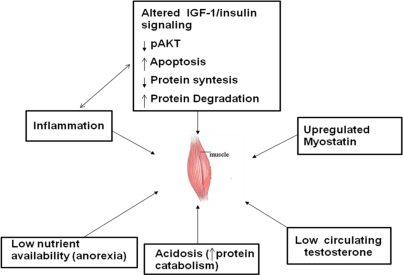
Scheme of major mechanisms promoting PEW in patients with chronic kidney disease.

In conclusion, PEW is common in patients with CKD and is associated with an increased death risk. However, while even minor renal dysfunction is an independent predictor of adverse cardiovascular prognosis, PEW becomes clinically manifest at an advanced stage, early before or during the dialytic stage. Mechanisms causing loss of muscle protein and fat are complex and not always associated with anorexia, but are linked to several abnormalities that stimulate protein degradation and/or decrease protein synthesis (Figure 2). In addition, data from experimental CKD indicate that uremia specifically blunts the regenerative potential in skeletal muscle, by acting on muscle stem cells. During the course of CKD, the loss of kidney excretory and metabolic functions proceeds together with the activation of pathways of endothelial damage, inflammation, acidosis, resistance to anabolic hormones, and defective insulin signaling. These factors may overlap those already operating in ageing and in comorbid conditions, such as diabetes and sepsis to orchestrate the PEW syndrome.
